# Response to Shepherd Comment on Mroczek et al. Evaluation of Quality of Life of Those Living Near a Wind Farm. *Int. J. Environ. Res. Public Health*, 2015, *12*, 6066–6083

**DOI:** 10.3390/ijerph14020141

**Published:** 2017-02-01

**Authors:** Bożena Mroczek, Joanna Banaś, Małgorzata Machowska-Szewczyk, Donata Kurpas

**Affiliations:** 1 Department of Humanities in Medicine, Faculty of Health Sciences, Pomeranian Medical University, 11 Chlapowskiego St., 70-204 Szczecin, Poland; 2 Faculty of Computer Science and Information Technology, West Pomeranian University of Technology, Szczecin Wydział Informatyki, 41 Zolnierska St., 71-210 Szczecin, Poland; jbanas@wi.zut.edu.pl (J.B.); mmachowska@wi.zut.edu.pl (M.M.-S.); 3 Department of Family Medicine, Wroclaw Medical University, 1 Syrokomli St., 51-141 Wroclaw, Poland; dkurpas@ hotmail.com; 4 Opole Medical School, 68 Katowicka St., 45-060 Opole, Poland

In response to the reservations, I would like to list the differences between the article published in *IJERPH* in 2015 [1] and the one published in AAEM in 2012 [2].

The article published in *IJERPH* presents an in-depth analysis of the results obtained in the study of 1277 people living in the vicinity of wind farms. In this article, the authors considered a greater number of environmental stress factors contributing to the quality of life (QoL) of residents. Aside from the distance between the wind farm and the place of residence, we took into account: stages of investment, the social acceptance of the investment in wind energy, and benefit derived from wind farms. We also analysed the influence of residents’ health problems, risky behaviours and stress-related problems on their QoL levels. We performed a multiple regression analysis to identify the strongest contributors to health status and QoL scores in eight SF-36 domains, as well as in the physical and mental components.

Additionally, we used a generalized linear model, determined the odds ratio (OR), and employed correspondence analysis to determine which environmental stress factors in the vicinity of wind-farm developments have the strongest effects on residents’ QoL and health status. Furthermore, we analysed the occurrence of mental health problems such as irritation, anxiety, anger with regard to environmental stress factors—the distance between residence and the wind turbines, and the stage of the development. These issues were not analysed in the article [2].

In the study [1] we did not analyse the influence of noise on the residents’ QoL and health status; therefore, information concerning these issues was limited and partially removed, as suggested by the *IJREPH* reviewers.

Regarding Omission One, we have never agreed with the widespread derision of the mentioned articles [3,4]. The report [3] is based on the studies of authors who are widely cited by other researchers, namely Pedersen E. (2007), van den Berg F, et al. (2008), Leventhall G., et al. (2003), Colby WD., et al. (2009) [5,6,7,8] and others.

We are familiar with the article of Onakpoya IJ., et al. (2015), but it concerns the influence of noise on sleep disorders, which was not a subject of our study [9]. We could not interpret our results with regard to bothersome noise generated by wind turbines, because we did not measure the noise levels near places of residence.

Regarding Omission Two, we analysed attitudes to new wind farm developments as one of the variables in our research project, and the nocebo effect was mentioned as the one most closely-related to attitudes. In our report [2] we raise the problem of honesty and justice associated with wind farm developments and their effects on attitudes and health problems, which requires further investigation and changes in law.

We did not refer to the findings by Poland’s Supreme Audit Office, because our purpose was to analyse health aspects of reactions to new developments in the environment on the example of wind turbines, and not to assess conduct of investors and politicians. Non-adherence to the standards of good practice in social consultations, and the lack of legal regulations for HIA caused the above-mentioned situations.

Regarding Omission Three, it is a precious remark; however, since the purpose of our study was to determine whether there exists a relationship between the presence of wind farms at different stages of development and the QoL of people living in their vicinity in Poland, we focused on the influence of selected factors on self-reported QoL.

As normative we regarded data which come from international studies and let us accept some norms for the assessment of QoL and health status [10].

Ware J.E., et al. (1995) proposed combining eight domains into two components: physical and mental ones [11]. “Results suggest that two summary measures may be useful in most studies and that their empiric validity, relative to the best SF-36 scale, will depend on the application. Survey offering the option of analyzing both a profile and psychometrically based summary measures have an advantage over health status assessment; health-related QOL; empiric validity, health index; factors analysis” [11]. In the analysis of the results, we combined the scores in the physical component, mental component, and the domains of general health and vitality in order to determine overall QoL. Comparison of overall QoL scores with physical and mental component scores helped us to determine which component had a stronger impact on the respondents’ QoL and health status. We believe it is not a mistake, especially that we gave all results that are needed to interpret the respondents’ QoL. We also compared effects of the variables analysed in the study on physical and mental component scores and separately on each of eight domains, which allowed us to draw the conclusions. We calculated an average overall QoL score to determine self-reported QoL—we did not regard it as a mistake but the statistical check for clarity of the results [11].

Regarding Omission Four, the study was assessed by three independent *IJERPH* reviewers, and we conformed to all their suggestions. The reviewers did not comment on methodology or purpose of our research. The study was based on the assumption that the stage of the wind farm development contributes to a subjective assessment of QoL and health status. In our opinion, the results obtained allow us to draw conclusions.

Regarding Omission Five, I do not agree that our findings are significantly different from the results achieved by the researchers quoted in the article, for example: Merlin T., et al. (2013), van den Berg F., et al. (2008), Pedersen E., et al. (2011, 2008, 2007, 2007a), Lombard A., et al. (2014), Johanson M., et al. (2007), Nissenbaum M., et al. (2011), Shepherd D., et al. (2010) [5,6,12,13,14,15,16,17,18,19].

The differences may result from the time when the study was conducted, cultural differences, and the fact that a movement against new developments began in Poland later than in other countries. So far, the results of randomized clinical research studies concerning a direct relationship between the occurrence of diseases and the distance between the wind farm and the place of residence have not been published. The minimum limit for a distance between wind farms and houses is different in various countries. In Poland it is more than 3 km, in other European countries 5 to 10 km [20].

Regarding Omission Six, i.e., the allegation concerning the conflict of interests. My name is Bożena Mroczek. I am the wife of Jarosław Mroczek, who is a CEO of EPA Ltd., which is an owner of multifarious companies. I declare, however, that my scientific research on the influence of wind farms on the health of people living in their vicinity has never been commissioned or financed by the EPA. I would also like to explain that the EPA has never been the owner of any wind farm. Its role is limited to the preparation of designs up to the stage of getting planning permission.

## Note from the Editor

This reply is the result of an extensive discussion with the academic editors. The discussion will also be published.

## References

[1] Mroczek B., Banaś J., Kurpas D., Machowska-Szewczyk M., Karakiewicz B. (2015). Evaluation of quality of life of those living near a wind farm. Int. J. Environ. Res. Public Health.

[2] Mroczek B., Kurpas D., Karakiewicz B. (2012). Influence of distances between places of residence and wind farms on the quality of life in nearby areas. Ann. Agric. Environ. Med..

[3] The Potential Health Impact of Wind Turbines (2010). Chief Medical Officer of Health (CMOH) Report. http://www.simcoemuskokahealth.org/Libraries/TOPIC_Environment/health_impacts_wind_turbines.sflb.ashx.

[4] Merlin T., Newton S., Ellery B., Milverton J., Farah C. (2013). Systematic Review of the Human Health Effects of Wind Farms.

[5] Pedersen E. (2007). Human Response to Wind Turbine Noise: Perception, Annoyance and Moderating Factors.

[6] Van den Berg F., Pedersen E., Bouma J., Bakker R. (2008). Project WINDFARMperception: Visual and Acoustic Impact of Wind Turbine Farms on Residents.

[7] Leventhall G., Pelmear P., Benton S. (2003). A Review of Published Research on Low Frequency Noise and Its Effects.

[8] Colby W.D., Dobie R., Leventhall G., Lipscomb D.M., McCunney R.J., Seilo M.T., Søndergaard B. (2009). Wind Turbine Sound and Health Effects. An Expert Panel Review.

[9] Onakpoya I.J., Sullivan J., Thompson M.J., Heneghana C.J. (2015). The effect of wind turbine noise on sleep and quality of life: A systematic review and meta-analysis of observational studies. Environ. Int..

[10] Maurish M.E. (2011). User’s Manual for the SF-36v2 Health Survey.

[11] Ware J.E., Kosinski M., Bayliss M.S., McHorney C.A., Rogers W.H., Raczek A. (1995). Comparison of methods for the scoring and statistical analysis of SF-36 health profile and summary measures: Summary of results from the medical outcomes study. Med. Care.

[12] Pedersen E., Hallberg L.R.-M., Waye K.P. (2007). Living in the vicinity of wind turbines: A grounded theory study. Qual. Res. Psychol..

[13] Pedersen E., Persson Waye K. (2007). Wind turbine noise, annoyance and self-reported health and well-being in different living environments. Occup. Environ. Med..

[14] Pedersen E., Larsman P. (2008). The impact of visual factors on noise annoyance among people living in the vicinity of wind turbines. J. Environ. Psychol..

[15] Pedersen E. (2011). Health aspects associated with wind turbine noise—Results from three field studies. Noise Control Eng. J..

[16] Lombard A., Ferreira S. (2014). Residents’ attitudes to proposed wind farms in the West Coast region of South Africa: A social perspective from the South. Energy Policy.

[17] Johanson M., Laike T. (2007). Intention to respond to local wind turbines: The role of attitudes and visual perception. Wind Energy.

[18] Shepherd D., Welch D., Dirks K.N., Mathews R. (2010). Exploring the relationship between noise sensitivity, annonyance and health-related quality of life in sample of adults exposed to environmental noise. Int. J. Environ. Res. Public Health.

[19] Nissenbaum M., Aramini J., Hanning C. (2011). Adverse health effects of industrial wind turbines: A preliminary report. Proceedings of the 10th International Congress on Noise as a Public Health Problem (ICBEN).

[20] Feder K., Michaud D.S., Keith S.E., Voicescu S.A., Marro L., Than J., Guay M., Denning A., Bower T.J., Lavigne E. (2015). An assessment of quality of life using the WHOQOL-BREF among participants living in the vicinity of wind turbines. Environ. Res..

